# A network psychometric evaluation of the Acceptance and Action Questionnaire – III and PsyFlex

**DOI:** 10.1371/journal.pone.0359352

**Published:** 2026-10-01

**Authors:** Francisco J. Ruiz, Douglas M. Bastidas-Suárez, Camilo A. Vargas

**Affiliations:** 1 Fundación Universitaria Konrad Lorenz, Bogotá, Colombia; 2 Universidad de San Buenaventura, Bogotá, Colombia; Universita degli Studi Europea di Roma, ITALY

## Abstract

Psychological flexibility and inflexibility are core processes in the Acceptance and Commitment Therapy (ACT) model and are consistently associated with well-being and emotional distress. However, the validity of widely used measures, particularly the Acceptance and Action Questionnaire – II (AAQ-II), has been questioned due to potential overlap with indicators of distress and neuroticism. This study examined the psychometric properties of the Acceptance and Action Questionnaire – III (AAQ-III) and the PsyFlex in a general population sample from Colombia (*N* = 785) using network psychometrics. We estimated the dimensionality of each scale via Exploratory Graph Analysis (EGA) and evaluated replicability via bootstrap EGA (bootEGA). Discriminant validity was examined using a joint EGA that included the AAQ-III, PsyFlex, and measures of emotional distress (DASS-21) and neuroticism (BFI-2-Neuroticism). Convergent validity was assessed through correlations with the Multidimensional Psychological Flexibility Inventory – Short Form. EGA identified unidimensional structures for both instruments, and bootEGA confirmed perfect structural consistency and item stability. The joint EGA yielded five distinct communities, with the AAQ-III and PsyFlex forming separate communities from emotional symptoms and neuroticism, providing structural evidence consistent with the discriminant validity of both measures. Both instruments showed the expected patterns of correlations with flexibility and inflexibility processes. These findings provide initial validation evidence that the Spanish versions of the AAQ-III and PsyFlex are psychometrically sound measures of psychological inflexibility and flexibility.

## Introduction

Acceptance and commitment therapy (ACT) is the most prominent application of contextual behavioral science (CBS) [1–3]. ACT proposes psychological flexibility as a major determinant of mental health, defined as “the ability to contact the present moment more fully as a conscious human being, and to change or persist in behavior when doing so serves valued ends” [4, p. 7]. This construct comprises six interrelated processes: acceptance, defusion, contact with the present moment, self-as-context, values, and committed action. A related and maladaptive behavioral pattern was termed psychological inflexibility, which is defined as the “rigid dominance of psychological reactions over chosen values and contingencies in guiding action” [5, p. 678] and comprises the counterpart of each flexibility process: experiential avoidance, cognitive fusion, lack of contact with the present moment, attachment to the conceptualized self, lack of values clarity, and inaction.

Meta-analyses and systematic reviews have shown that both constructs are relevant transdiagnostic processes, to the extent that increases in psychological flexibility are associated with reductions in emotional symptoms and improvements in quality of life and behavioral effectiveness, whereas psychological inflexibility is associated with psychopathology and impaired functioning [6–9]. Importantly, measures of psychological flexibility and inflexibility show differential patterns of correlation with related constructs: flexibility measures tend to correlate more strongly with positive constructs (e.g., quality of life), whereas inflexibility measures tend to correlate more strongly with negative constructs (e.g., depression, anxiety) [10,11]. This evidence suggests that psychological flexibility and inflexibility might be related but distinct functional patterns rather than exact opposites [12]. However, most of this evidence comes from studies using multidimensional instruments that assess flexibility and inflexibility processes on separate scales, and the degree of overlap between these constructs when measured with brief, unidimensional instruments remains an open question.

The measurement of psychological flexibility and inflexibility has followed two main approaches: (a) unidimensional instruments that assess psychological flexibility or inflexibility as a single construct, and (b) multidimensional instruments that assess their component processes separately (e.g., the Multidimensional Psychological Flexibility Inventory, MPFI) [12] or grouped into broader dimensions (e.g., the CompACT) [13]. This study focuses on unidimensional instruments given their practical advantages for clinical assessment, screening, and research purposes.

The Acceptance and Action Questionnaire – II (AAQ-II) [5] has been the most widely used self-report measure of psychological inflexibility in ACT research during the last fifteen years. The AAQ-II consists of seven items that reflect psychological inflexibility, although it has been inconsistently used as a measure of psychological inflexibility, experiential avoidance, and psychological flexibility, which has generated considerable confusion [14,15]. The AAQ-II has accumulated evidence of good to excellent internal consistency, test-retest reliability, and a generally stable one-factor structure that has proven to be invariant across gender, clinical and nonclinical samples, and cultures [5,16,17]. Additionally, the AAQ-II has demonstrated incremental validity in predicting outcomes above and beyond neuroticism and emotional symptoms (e.g., [18,19],).

However, some recent studies have identified concerns about its discriminant validity, suggesting that the AAQ-II may primarily measure trait negative emotion or neuroticism rather than psychological inflexibility [14,20,21]. Ruiz et al. [15] argued that most discriminant validity issues stem from the inconsistent use of the AAQ-II as a measure of psychological flexibility and experiential avoidance rather than psychological inflexibility and, using exploratory graph analysis (EGA) [22], showed that the AAQ-II items formed a distinct community separate from measures of emotional symptoms and neuroticism.

These debates motivated the development of refined instruments. The Acceptance and Action Questionnaire – III (AAQ-III) [23,24] was developed to address the limitations in discriminant validity identified in the AAQ-II. Item response theory analyses of the AAQ-II identified problems with semantic clarity, variability in item functioning across samples, and suboptimal discrimination parameters. The AAQ-III revised the wording of all seven AAQ-II items to improve clarity and specify the functional consequences of psychological inflexibility. For example, the vague AAQ-II item “I’m afraid of my feelings” was reworded to “I’m so afraid of my feelings that I don’t do things I care about,” making explicit the behavioral impact of experiential avoidance. Similarly, “Emotions cause problems in my life” was changed to “How I react to emotions causes problems in important areas of my life,” shifting the focus from emotions themselves to one’s response to them. The AAQ-II item “It seems like most people are handling their lives better than I am,” which introduced social comparison rather than measuring psychological inflexibility per se, was replaced with the more functionally specific “I do not handle my emotions well.” Ong et al. [24] compared the AAQ-II, the AAQ-III, the Brief Experiential Avoidance Questionnaire (BEAQ) [25], and the CompACT [13] in community (n = 253), student (n = 261), and treatment-seeking (n = 140) samples using exploratory factor analyses and multigroup graded-response models. In that study, the AAQ-III showed stronger discriminant validity from measures of negative affect than the AAQ-II and the BEAQ, with the CompACT showing the strongest differentiation. To date, the only cross-cultural adaptation available is the Chinese version [26]. In a sample of 1,572 college students, the Chinese AAQ-III confirmed the one-factor structure of the original version through exploratory and confirmatory factor analyses, showed good internal consistency and measurement invariance across gender, and demonstrated better convergent and discriminant validity than the AAQ-II and the BEAQ.

Regarding the assessment of psychological flexibility through a unidimensional instrument, Gloster et al. [27] developed the Psychological Flexibility Scale (PsyFlex). The PsyFlex comprises six items, each representing one process from the hexaflex model. A distinctive feature of this instrument is its context-sensitive design: items refer to the respondent’s experience during the last seven days, which contrasts with trait-like measures (e.g., the AAQ-II). The PsyFlex was initially validated with four English-speaking samples (*N* = 744), including community, nonclinical, and clinical samples. Confirmatory factor analysis confirmed a one-factor structure with excellent reliability and measurement invariance between clinical and nonclinical samples. Additionally, the PsyFlex demonstrated good convergent validity with measures of psychological flexibility and symptomatology, and discriminated between clinical and nonclinical groups [27]. Subsequent cross-cultural validation studies have replicated these findings: the European Portuguese version reproduced the one-factor structure and the expected associations with mental health in adults [28]; the Korean version was validated in a community sample [29]; the Turkish version showed the expected associations with resilience and social support [30]; the Hebrew version replicated the psychometric properties of the original version [31]; and the Chinese version extended the evidence to parents of children with autism spectrum disorder [32].

An important advantage of the PsyFlex is its treatment sensitivity, given that Benoy et al. [33] found that the AAQ-II showed lower treatment sensitivity than context-sensitive measures of psychological flexibility, suggesting that treatment effects on psychological flexibility may have been underestimated in studies relying on trait-like instruments. Given its temporally specific design, the PsyFlex is particularly suited for repeated administrations aimed at capturing treatment-related change. In Spanish-speaking contexts, the assessment of psychological (in)flexibility has relied predominantly on the AAQ-II [17,34,35], and no studies have yet examined the psychometric properties of the AAQ-III in these populations, and the evidence on the PsyFlex remains scarce. Accordingly, this study aimed to analyze the psychometric properties of the Spanish versions of the AAQ-III and PsyFlex using network psychometric methods. We adopted EGA [22] as the primary analytic strategy, given that it does not rely on latent-variable model assumptions, which is consistent with the functional contextualist standpoint of CBS, which emphasizes the usefulness of measures in advancing toward prediction-and-influence goals rather than assuming they reflect pre-organized realities [36,37]. Additionally, EGA reduces some researcher degrees of freedom, given that its algorithm is deterministic once the estimation method and the community detection algorithm have been specified [15]. We regard network psychometrics as a complement to factor-analytic approaches rather than as a substitute for them. Specifically, we first analyzed the dimensionality and stability of both instruments independently through EGA and bootstrap EGA (bootEGA) [38]. Second, we examined evidence of discriminant validity by conducting a joint EGA that included these instruments along with measures of emotional symptoms (i.e., the Depression Anxiety and Stress Scales – 21, DASS-21) [39] and neuroticism (i.e., the Neuroticism subscale of the Big Five Inventory – 2, BFI-2) [40]. Lastly, we assessed convergent validity through correlations with the Multidimensional Psychological Flexibility Inventory – Short Form (MPFI-SF) [12] and the Satisfaction with Life Scale (SWLS) [41].

## Materials and methods

### Participants

The sample consisted of 785 participants residing in Colombia, with an average age of 30.10 years (*SD* = 11.32). Most participants were female (61.40%), while 37.96% were male, and five participants (0.64%) chose not to disclose their gender. Regarding socioeconomic status, most participants were classified as low (54.40%), 43.57% as medium, and only 2.04% as high. In terms of educational attainment, 1.78% had completed primary education, 30.70% had finished high school, 40.64% were technicians, 18.47% held college degrees, and 8.41% pursued postgraduate education. Concerning civil status, 61.78% of participants were single, 35.67% were married or in a free union, 1.66% were divorced, and 0.89% were widowed. At the time of the survey, only 9.81% were currently receiving psychological or psychiatric treatment. In comparison, 26.62% reported having received some form of psychological or psychiatric treatment at some point in their lives.

### Instruments

***Acceptance and Action Questionnaire – III*** (AAQ-III) [23,24]. The AAQ-III is a self-report measure of psychological inflexibility comprising 7 items rated on a 7-point Likert scale (1 = *never true*, 7 = *always true*). It has demonstrated a one-factor structure, excellent internal consistency, and better discriminant validity than its predecessor (i.e., the AAQ-II).

***PsyFlex*** [27]. This self-report instrument assesses psychological flexibility using six items, each representing a process from the hexaflex model of psychological flexibility. Participants respond using a five-point Likert scale from 1 (*very seldom*) to 5 (*very often*). The PsyFlex has demonstrated a one-factor structure and convergent validity with several instruments that measure psychological (in)flexibility or related processes.

***Multidimensional Psychological Flexibility Inventory – Short Form*** (MPFI-SF) [12,42]. The MPFI-SF is a 24-item scale designed to assess psychological flexibility and inflexibility over the past two weeks. This scale was developed from the 60-item MPFI using item response theory analyses to select the two most effective items from each subscale. Participants rate items on a 6-point, Likert-type scale (1 = *never true*, 6 = *always true*). The MPFI-24 maintains the same factor structure as the original MPFI, comprising twelve first-order factors that represent each process of the hexaflex model, organized into two second-order factors that represent psychological flexibility and inflexibility. The MPFI is recognized as one of the most comprehensive and psychometrically sound instruments for measuring psychological flexibility [14]. The MPFI and MPFI-24 have shown good psychometric properties in Colombia and other Spanish-speaking countries [42]. The psychological flexibility composite, which includes all six flexibility processes, achieved an alpha of  .94 in this study. The inflexibility composite, comprising all six inflexibility processes, had an alpha of  .91.

***Big Five Inventory-2 – Neuroticism Subscale*** (BFI-2) [40], Spanish version by Gallardo-Pujol et al. [43]. The BFI-2 is a self-report instrument with 60 items, which participants answer using a 5-point Likert scale (1 = *strongly disagree*; 5 = *strongly agree*). It assesses the personality traits outlined by the Big Five model [44]. The BFI-2 has shown good internal consistency and has the expected five-factor structure in its validation study. The Spanish version of this measure also exhibits strong psychometric properties. In this study, we administered only the Neuroticism subscale (BFI-NE, also known as Negative Emotionality), comprising 12 items. The BFI-NE subscale obtained an alpha coefficient of  .82.

***Depression Anxiety and Stress Scales – 21*** (DASS-21) [39], Spanish version by Daza et al. [45]. The DASS-21 is a widely used self-report instrument consisting of 21 items, rated on a 4-point Likert scale (0 = did not apply to me at all; 3 = applied to me very much or most of the time). It includes three subscales: Depression, Anxiety, and Stress. The Spanish version of the DASS-21 has demonstrated strong internal consistency and a hierarchical factor structure, featuring a second-order factor representing a general measure of emotional symptoms and three first-order factors for each subscale [46]. The overall DASS-21 scale had an alpha of  .96, while the subscale alphas were  .91 for Depression and Anxiety, and  .90 for Stress.

***Satisfaction with Life Scale*** (SWLS) [41], Spanish translation by Atienza et al. [47]. The SWLS is a 5-item scale that uses a 7-point Likert scale (1 = *strongly disagree*, 7 = *strongly agree*) to measure self-perceived well-being. The Spanish version of the SWLS has shown good psychometric properties, a one-factor structure, and convergent validity in Colombia [48]. In this study, the SWLS had a Cronbach’s alpha of  .89.

### Procedure

The Institutional Ethics Committee of Fundación Universitaria Konrad Lorenz approved the study procedures (No. 25). The study was conducted between 10^th^ April and 24^th^ November 2023. The AAQ-III and PsyFlex translations were informed by the guidelines of Muñiz et al. [49]. The translation of the AAQ-III followed a forward-translation procedure with expert-panel reconciliation. Two ACT researchers with bilingual proficiency in Spanish and English independently translated the seven AAQ-III items. Both translators used the Spanish version of the AAQ-II developed by Ruiz et al. [35] as the starting point, which has accumulated evidence of construct validity in Colombia and other Spanish-speaking countries [17,34]. This decision was made because the AAQ-III revised the wording of the AAQ-II items rather than introducing entirely new content, allowing translators to preserve semantic continuity for items whose meaning had not changed and to focus on the functional specifications introduced in the AAQ-III. The two independent translations were subsequently reviewed by a panel of four researchers with expertise in ACT and CBS, who discussed each item until consensus was reached on the version that best preserved the semantic content, the functional specification, and the cultural appropriateness of each item.

Regarding the PsyFlex, this study did not generate a new translation. The starting point was the Spanish version previously developed by members of our research team for the international study on the mental health impact of the COVID-19 pandemic conducted by Gloster et al. [50]. Although this version had been used in prior research, its psychometric properties had not been formally examined in Spanish-speaking samples at the time of this study. For this study, the same expert panel reviewed each item against the original English wording and introduced modifications to items whose wording could be improved for fluency, semantic precision, or alignment with the hexaflex process represented by the item. The final wording of the items of both instruments is shown in Tables 1 and 2.

**Table 1:** Item translation into Spanish, original English version, and revised network loadings of the AAQ-III items.

Item	Network loading
1. Las preocupaciones, sentimientos y recuerdos dolorosos me impiden vivir una vida significativa [Painful worries, feelings, or memories make it impossible for me to live a meaningful life]	.48
2. Tengo tanto miedo de mis sentimientos que no puedo hacer las cosas que me importan [I’m so afraid of my feelings that I don’t do things I care about]	.53
3. Me preocupa perder el control de mis pensamientos, sentimientos y recuerdos [I worry about losing control of my thoughts, feelings, or memories]	.41
4. Los recuerdos dolorosos me impiden tener una vida plena [Painful memories prevent me from having a fulfilling life]	.53
5. La forma en la que reacciono ante mis emociones causa problemas en las áreas importantes de mi vida [How I react to emotions causes problems in important areas of my life]	.54
6. No manejo bien mis emociones [I do not handle my emotions well]	.40
7. Las preocupaciones, sentimientos y recuerdos me impiden avanzar en mis objetivos [Worries, feelings, or memories keep me from moving toward my goals]	.57

**Table 2:** Item translation into Spanish, original English version, and revised network loadings of the PsyFlex items.

Item	Network loading
1. Puedo concentrarme en lo que ocurre en los momentos importantes, incluso cuando aparecen pensamientos que podrían distraerme [Even if I am somewhere else with my thoughts, I can focus on what’s going on in important moments]	.51
2. Si es necesario, puedo dejar que los pensamientos y experiencias desagradables aparezcan sin tener que eliminarlos [If need be, I can let unpleasant thoughts and experiences happen without having to get rid of them immediately]	.31
3. Puedo observar los pensamientos difíciles desde cierta distancia, sin dejar que me controlen [I can look at hindering thoughts from a distance without letting them control me]	.56
4. Incluso cuando los pensamientos y experiencias me alteran, puedo notar una parte interna en mí que es estable [Even if thoughts and experiences are confusing me I can notice something like a steady core inside of me]	.51
5. Yo determino qué es importante para mí y decido cómo quiero invertir mi energía [I determine what’s important for me and decide what I want to use my energy for]	.56
6. Me comprometo activamente con las cosas que son importantes, útiles o significativas para mí [I engage thoroughly in things that are important, useful, or meaningful to me]	.44

These translated instruments were uploaded to a Google Form that also included a section on written informed consent and a sociodemographic questionnaire. The study’s purpose was clearly explained, and participants consented to participate and to respond anonymously. The survey was shared via social media, and participants received no compensation for their participation.

### Data analysis

All statistical analyses were performed in R [51] using the package EGAnet [52]. We adopted exploratory graph analysis (EGA) [22] as our primary analytic strategy. EGA is a network psychometric technique that identifies latent dimensions as communities of nodes that are more densely connected among themselves than with the rest of the network. EGA differs from traditional factor analytic methods in several respects: it does not assume the latent variable model, involves fewer subjective decisions once the estimation method and the community detection algorithm have been specified, and simulation studies have found that it has comparable or better accuracy than parallel analysis in determining the number of dimensions [38,53].

Network estimation was conducted through the graphical least absolute shrinkage and selection operator (GLASSO) [54], with model selection based on the Extended Bayesian Information Criterion (EBIC) [55]. The tuning hyperparameter was set to *γ* = .50 following Christensen and Golino [38]. Once the partial-correlation network was estimated, items were grouped into communities using the Louvain algorithm, which has shown high accuracy and minimal bias in reconstructing underlying patterns in simulation studies [56].

We first analyzed the dimensional structure of the AAQ-III and PsyFlex independently. Revised network loadings [57] were computed to assess the relative contribution of each item to its designated dimension. These loadings are analogous to factor loadings in latent variable models. Following conventional criteria, loadings of  .50 or higher were considered strong, values between  .35 and  .50 moderate, values between  .20 and  .34 weak, and values below  .20 poor.

Subsequently, we computed bootstrap EGA (bootEGA) [38] with 500 parametric bootstrap samples to assess the stability and reproducibility of the community solutions. BootEGA generates a sampling distribution of EGA results and provides (a) the median number of dimensions along with 95% confidence intervals, (b) structural consistency (the percentage of bootstrap samples replicating the same structure), and (c) item stability (the percentage of times each item is assigned to the same dimension). Values ≥.75 for structural consistency and ≥.70 for item stability were considered indicators of adequate stability [38,58].

To evaluate discriminant validity, we conducted a combined EGA that included the AAQ-III, PsyFlex, DASS-21, and the Neuroticism subscale of the BFI-2. According to this approach, clear separation between flexibility measures and distress item communities provides item-level evidence consistent with discriminant validity, although this criterion is not equivalent to conventional tests based on latent variable models [15,53]. Lastly, we computed Pearson correlations between the AAQ-III and PsyFlex and the remaining instruments to assess convergent validity.

## Results

### EGA and bootEGA of the AAQ-III

The results of the EGA performed with the AAQ-III are displayed in the left panel of Fig 1, demonstrating a unidimensional structure in which all items form a single community (TEFI = 0). Table 1 shows that the standardized network loadings ranged from  .40 to  .57, indicating moderate to strong relationships among the items. The highest loadings were found for items 7 (.57), 5 (.54), 4 (.53), and 2 (.53), highlighting that these items are the most pivotal within the network.

**Fig 1:**
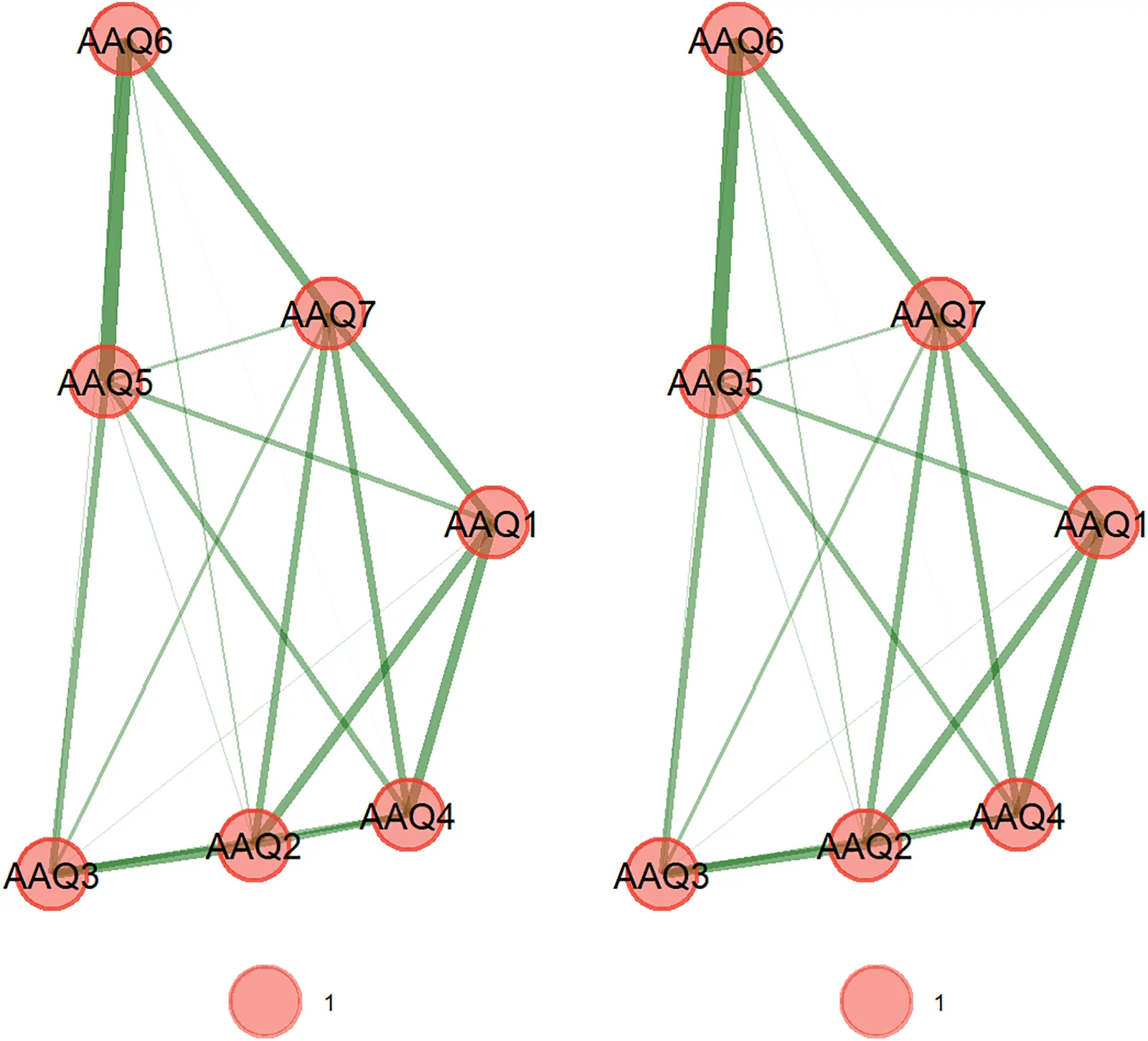
Networks resulting from EGA (left) and bootEGA (right) of the AAQ-III.

The results of the bootEGA applied to the AAQ-III items are displayed in the right panel of Fig 1. The bootEGA validated the stability of this unidimensional solution. In all bootstrap samples, the one-factor solution was identified 100% of the time, with structural consistency and item stability both reaching a maximum of 1.00. Overall, these findings indicate that the AAQ-III consistently maintains a stable, coherent, and replicable unidimensional structure across resampled data.

### EGA and bootEGA of the PsyFlex

The left panel of Fig 2 presents the results of the EGA conducted with the PsyFlex, which demonstrated a unidimensional structure (TEFI = 0), where all items were associated with a single community. Standardized network loadings ranged from  .31 to  .56, indicating moderate to high item coherence (see Table 2). The items with the highest loadings were items 3 (.56) and 5 (.56), implying that these items are crucial in shaping the community.

**Fig 2:**
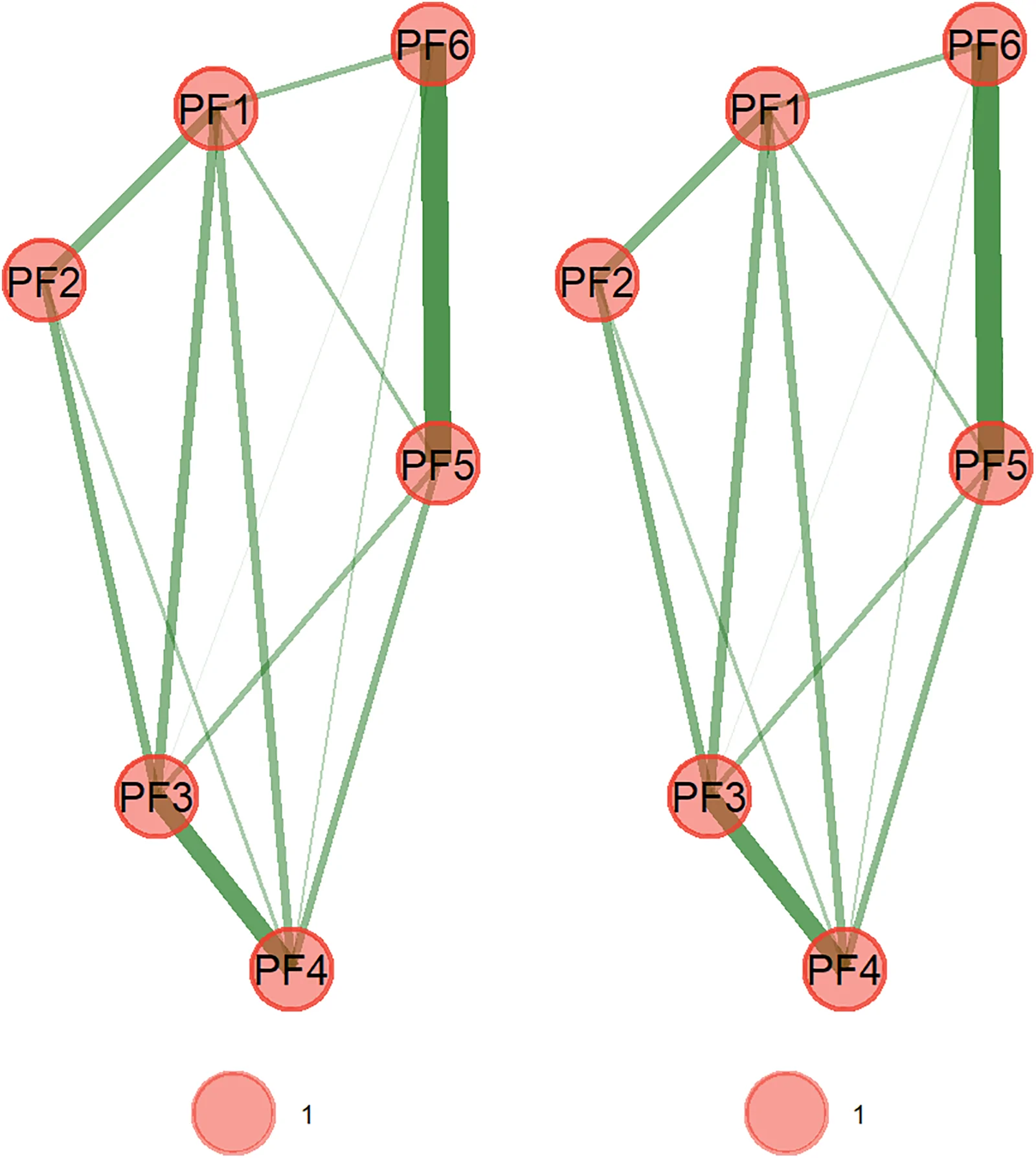
Results of the EGA (left panel) and bootEGA (right panel) of the PsyFlex.

The bootEGA confirmed the reliability of this unidimensional solution across all samples (see Fig 2, right panel). The structural consistency and item stability were both measured at 1.00, confirming the dependability of the dimensional arrangement. Collectively, these results suggest that the PsyFlex scale exhibits a strong, reproducible unidimensional structure, characterized by high item coherence and perfect stability across the samples.

### Discriminant validity analysis

Fig 3 shows that the joint EGA, including the AAQ-III, PsyFlex, DASS-21, and BFI-2-Neuroticism, yielded five distinct communities (TEFI = −59.17). The AAQ-III and PsyFlex items formed independent communities separate from emotional symptoms and neuroticism. All items of the DASS-21 were grouped in a single community, whereas the items of the BFI-2-Neuroticism were grouped in two communities.

**Fig 3:**
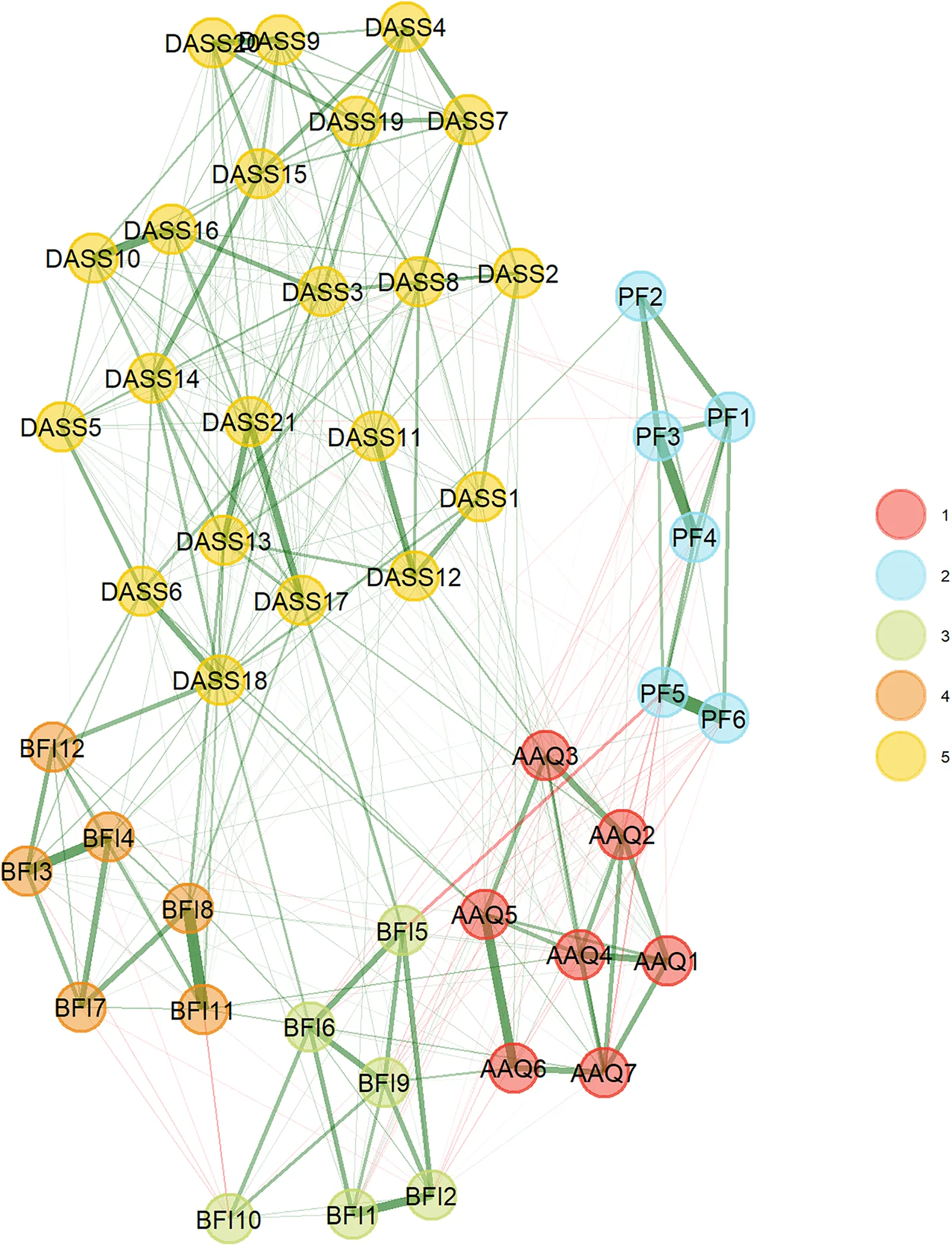
Results of the EGA conducted with the AAQ-III, PsyFlex, DASS-21, and BFI-2-Neuroticism.

The bootEGA provided further evidence of the stability of this configuration, comprising five dimensions (see Fig 4). The most common solution involved five dimensions (76.4% of samples), followed by six dimensions (16.8%) and four dimensions (6.8%), with a median of 5 and a 95% confidence interval of [4.07, 5.93]. The coefficients for structural consistency ranged from  .73 to 1.00, with the AAQ-III and PsyFlex exhibiting perfect structural consistency.

**Fig 4:**
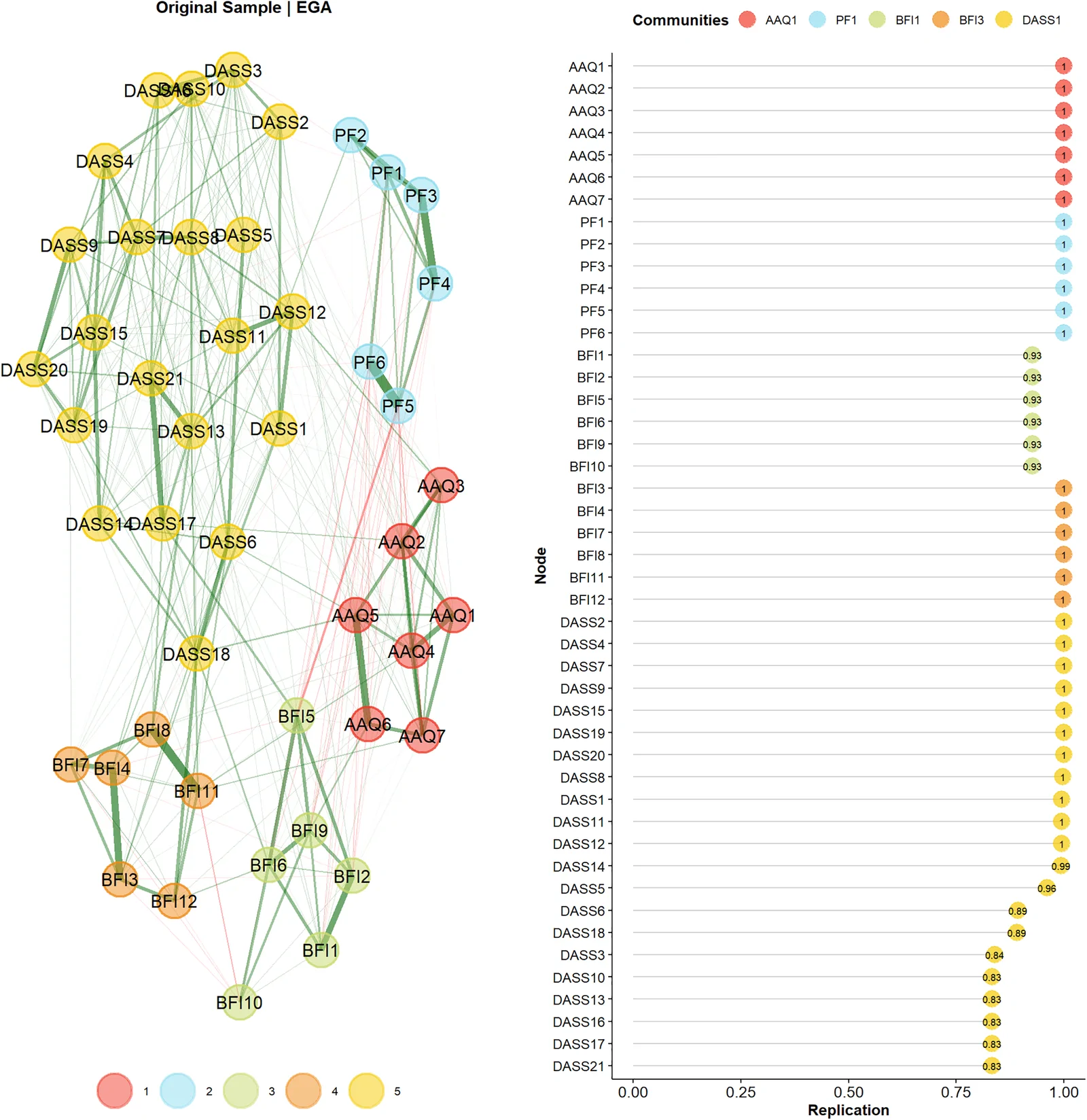
Results of the bootEGA conducted with the AAQ-III, PsyFlex, DASS-21, and BFI-2-Neuroticism.

When examining individual items, all AAQ-III and PsyFlex items showed perfect stability (1.00), whereas items from the DASS-21 and BFI-2-Neuroticism also showed good stability (ranging from  .83 to 1.00). These results strongly support the discriminant validity of the AAQ-III and PsyFlex in comparison to measures of emotional symptoms and neuroticism.

### Correlations between the AAQ-III, PsyFlex, and related instruments

Fig 5 shows a heatmap illustrating the correlation matrix among all study variables. The AAQ-III showed strong positive correlations with indicators of psychological distress and some processes of the hexaflex model, such as cognitive fusion, self-as-content, inaction, and lack of values clarity. It also displayed moderate negative correlations with adaptive ACT-related processes, including acceptance, present-moment awareness, self-as-context, defusion, values, and committed action. This pattern suggests that higher psychological inflexibility, as measured by the AAQ-III, is consistently associated with poorer mental health and lower engagement in ACT processes.

**Fig 5:**
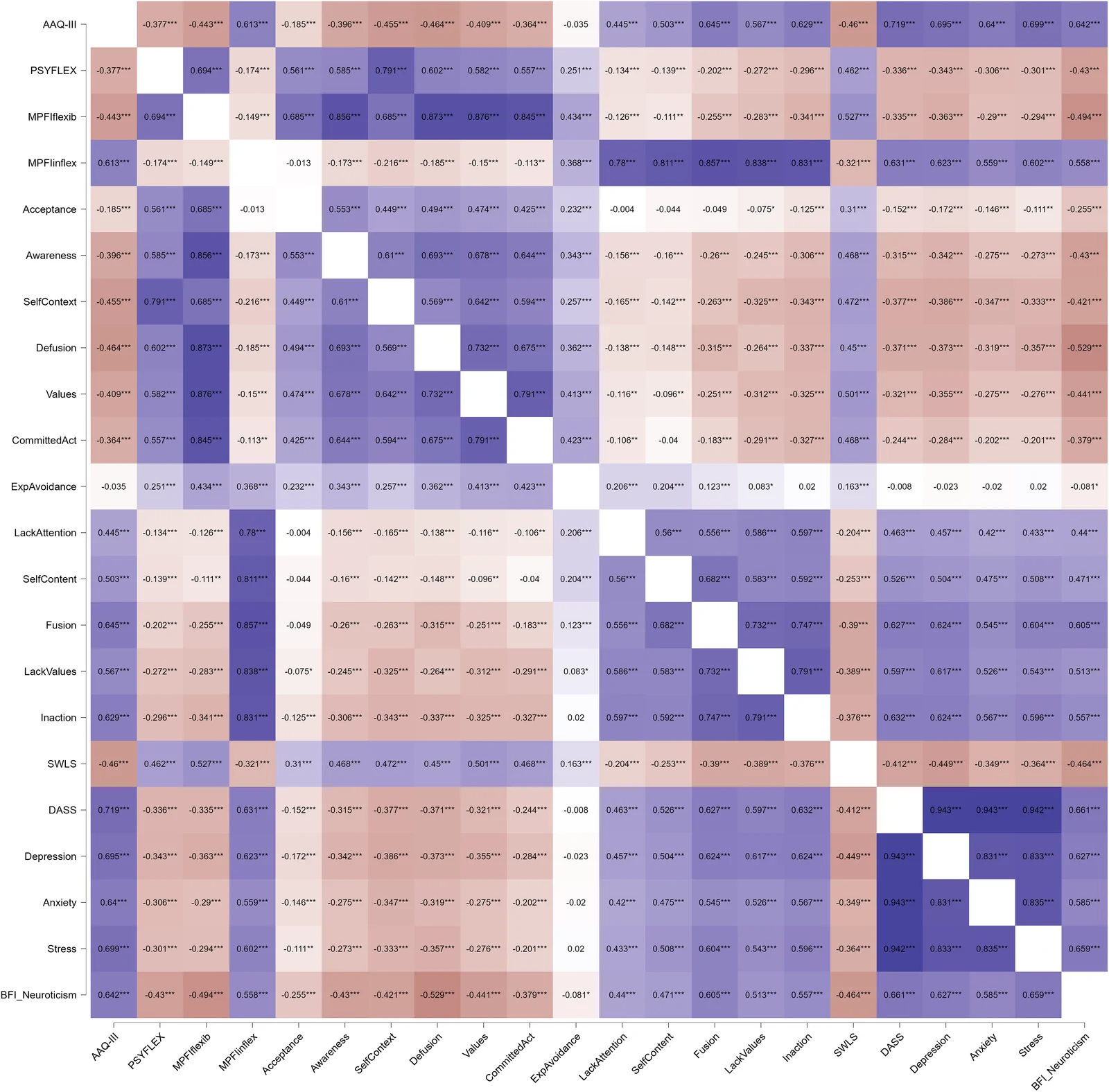
Heatmap of the correlations of the AAQ-III and PsyFlex with the remaining instruments.

The PsyFlex, in contrast, showed the opposite correlation pattern. It correlated positively with psychological flexibility processes (acceptance, present-moment awareness, self-as-context, defusion, values, and committed action) and negatively with cognitive fusion, inaction, lack of values, and psychological distress measures. These correlations indicate that PsyFlex serves as a robust index of psychological flexibility, aligning with protective mental health processes and lower symptomatology.

Both instruments were associated with neuroticism and in the expected direction: AAQ-III correlated positively, whereas PsyFlex correlated negatively. Lastly, as expected, the AAQ-III and PsyFlex demonstrated a moderate negative correlation (*r* = −.38).

## Discussion

The current study aimed to analyze the psychometric properties of two brief measures of psychological flexibility and inflexibility, the AAQ-III and the PsyFlex, in a Colombian general population sample. To date, the assessment of these constructs in Spanish-speaking contexts has predominantly relied on the AAQ-II, a measure that has been criticized for its lack of discriminant validity from neuroticism and emotional distress [14,59]. The results of this study provide initial evidence supporting the internal structure, stability, and validity of both measures using network psychometric methods.

The EGA and bootEGA analyses confirmed the unidimensionality of both the AAQ-III and the PsyFlex. The one-dimensional solution was replicated in 100% of the bootstrap samples for both instruments, with perfect item stability and structural consistency. For the AAQ-III, these findings are consistent with the original validation study by Ong et al. [24] and indicate that the seven items cohesively represent the broader construct of psychological inflexibility. The standardized network loadings ranged from  .41 to  .57, with the highest values corresponding to items 7, 5, 4, and 2. These items describe the interference that distressing emotions and memories cause in meaningful life and goal achievement, as well as the maladaptive emotional responses they elicit. This pattern supports conceptualizing the AAQ-III as a measure of overall psychological inflexibility, in which cognitive fusion and experiential avoidance are captured through their functional impact on daily living.

Similarly, the PsyFlex showed a robust unidimensional structure, replicating the findings of Gloster et al. [27]. Network loadings ranged from  .31 to  .56, with the highest values for items 3 (defusion) and 5 (values). These results suggest that, in the Colombian context, psychological flexibility operates as a unified construct in which acceptance, defusion, present-moment awareness, self-as-context, values, and committed action form a tightly interconnected network. The remarkable stability observed in the bootEGA further supports the use of the PsyFlex as a brief and reliable measure of psychological flexibility for Spanish-speaking populations.

A central finding of this study concerns the structural differentiation between these measures and related constructs. The combined EGA, which included the AAQ-III, PsyFlex, DASS-21, and BFI-2-Neuroticism, yielded five distinct communities. Unlike the typical pattern reported for the AAQ-II in previous studies, in which items frequently cluster with stress or anxiety factors [20,21], the AAQ-III formed an independent community, clearly separated from emotional symptoms and personality traits. The same was observed for the PsyFlex. The grouping of all DASS-21 items into a single community is consistent with evidence supporting a general factor of negative affect or emotional distress [46]. The bifurcation of neuroticism into two communities likely reflects a wording effect due to reverse-scored items, a pattern commonly observed in factor-analytic studies of the BFI-2 [40].

The perfect stability of the AAQ-III and PsyFlex communities across bootstrap samples strengthens the conclusion that these measures assess distinct and replicable dimensions in this cultural context. These findings are consistent with previous evidence indicating that the AAQ-III shows better discriminant validity than the AAQ-II [15,24] and suggest that both instruments capture functional behavioral processes that are distinguishable from symptom reporting or neuroticism. However, given that the AAQ-II was not administered in the current study, this comparison is indirect and rests on patterns reported in previous research. This differentiation is clinically relevant because it enables practitioners to assess whether treatment modifies how individuals relate to their experiences (i.e., flexibility) rather than merely the intensity of those experiences.

Regarding convergent validity, the correlation patterns with the MPFI-SF provided solid theoretical support. The AAQ-III showed strong positive correlations with the inflexibility composite and its subprocesses, whereas the PsyFlex correlated strongly with the flexibility composite and processes such as acceptance and committed action. The AAQ-III and PsyFlex showed a moderate correlation (r = −.38), indicating that the two measures are not interchangeable. The AAQ-III appears particularly suited for identifying behavioral rigidity and problematic cognitive fusion, showing stronger associations with depression, anxiety, and stress. Conversely, the PsyFlex better captures adaptive repertoires, exhibiting positive links with openness and engagement processes. Overall, these findings suggest that the PsyFlex functions as a marker of protective psychological resources, while the AAQ-III operates as an indicator of transdiagnostic vulnerability. They also provide evidence, based on brief unidimensional instruments, for the view that flexibility and inflexibility are related but distinguishable functional patterns, a question that had been examined mainly through multidimensional measures such as the MPFI.

Two additional patterns of the correlation matrix warrant conceptual comment. First, the AAQ-III correlated more strongly with the DASS-21 (r = .72) than with the inflexibility composite of the MPFI-SF (r = .61) and its specific factors (rs ranging from −.04 to  .65). We think this pattern reflects the item content of the AAQ-III, which emphasizes the interference of distressing private experiences with valued living and, therefore, shares affective content with symptom measures that is less present in the process-specific wording of the MPFI-SF. In addition, psychological inflexibility is conceptualized as a process directly involved in generating and maintaining emotional distress, to the extent that strong correlations with symptom measures are theoretically expected. Importantly, the joint EGA indicates that this overlap does not entail item-level redundancy: the AAQ-III items formed a community clearly separated from emotional symptoms and neuroticism. The near-zero correlation of the AAQ-III with the experiential avoidance factor of the MPFI-SF, which also showed positive correlations with flexibility processes, is consistent with the theoretically inconsistent correlations that this factor has shown in previous validations of the MPFI and that have been identified as one of the clear weaknesses of the instrument [60–62]. This pattern suggests that self-reports of deliberate avoidance strategies might capture a different behavioral pattern in nonclinical samples.

Second, the positive correlation of the AAQ-III with the DASS-21 (r = .72) was substantially stronger than the negative correlation of the PsyFlex (r = −.34), which is consistent with the view that both instruments measure related but not opposite processes. However, this asymmetry was not observed for life satisfaction. The correlations of the AAQ-III and the PsyFlex with the SWLS were of similar magnitude (rs = −.46 and  .46, respectively), which qualifies the differential pattern described above: whereas the AAQ-III was more closely tied to emotional symptoms, both instruments related comparably to global life satisfaction. A plausible interpretation is that life satisfaction judgments are sensitive both to the presence of emotional distress and to engagement in valued living, so that the vulnerability pole (AAQ-III) and the resources pole (PsyFlex) contribute to them through different paths. Accordingly, the characterization of the PsyFlex as a marker of protective resources and the AAQ-III as an indicator of transdiagnostic vulnerability applies more clearly to symptom measures than to global well-being indicators. These differential patterns might be examined in future studies including measures of valued living and positive functioning beyond global life satisfaction.

While this article was under review, two studies examined the psychometric properties of Spanish versions of the PsyFlex in Spain. Navarrete et al. [61] analyzed a different Spanish translation in individuals with chronic pain, whereas Crego et al. [63] analyzed the same version used in the current study in a sample of 170 healthy individuals, finding a one-factor structure, good internal consistency, and high test-retest reliability. The current study complements this emerging evidence by providing the first examination of the PsyFlex in a large general population sample from Latin America and the first psychometric analysis of the Spanish AAQ-III.

Some limitations of this study warrant mention. First, the sample was collected through online convenience sampling and was predominantly female, which may limit the generalizability of the findings to the broader Colombian population or other cultural contexts. Second, all data were self-reported and collected cross-sectionally, precluding causal inference and potentially introducing common method variance. Third, the study did not include diagnostic interviews or behavioral assessments; thus, criterion validity with respect to clinical diagnoses or objective functioning indicators remains to be examined. Fourth, although the unidimensional solutions were highly consistent, measurement invariance across sex, age, and cultural context, as well as test-retest reliability, should be evaluated in future research. Fifth, although the translation procedures included two independent forward translations for the AAQ-III and an expert panel review for both instruments, formal back-translation by an independent bilingual translator was not implemented, and the items were not subjected to cognitive interviewing with members of the target population before administration; both procedures are recommended in the guidelines for test translation and adaptation [49] and should be incorporated in future cross-cultural validation work. Lastly, the evidence of discriminant validity provided by the joint EGA is structural in nature: the separation of item communities indicates that the AAQ-III and PsyFlex items share more unique variance among themselves than with indicators of emotional distress and neuroticism, but this criterion is not equivalent to conventional standards based on latent variable models (e.g., correlations between latent factors or heterotrait-monotrait ratios). Accordingly, our conclusions regarding discriminant validity should be interpreted as complementary to evidence derived from latent variable approaches. A related limitation is that this study did not include the AAQ-II, which precludes direct comparative analyses between the AAQ-III and its predecessor.

Further studies might extend these findings to clinical populations and other Spanish-speaking countries, examine longitudinal models that assess the predictive roles of psychological flexibility and inflexibility in symptom trajectories and quality of life, and incorporate multi-method assessments, such as third-party evaluations, behavioral tasks, and physiological measures. The network loading results indicate that specific items are particularly central within each construct, which could inform the development of ultra-brief versions or tailored adaptations for clinical or research purposes.

In conclusion, this study provides initial evidence of validation for the AAQ-III and PsyFlex in Colombia using network psychometrics. Both instruments demonstrated stable unidimensional structures and, importantly, formed item communities that were clearly separated from those of emotional distress and neuroticism. The availability of these validated measures offers clinicians and researchers in Colombia brief, reliable options for assessing core ACT processes in Spanish-speaking contexts.
