# Supplementary material for: A network psychometric evaluation of the Acceptance and Action Questionnaire – III and PsyFlex
Source: PLoS One. 2026 Oct 1;21(10):e0359352. doi: 10.1371/journal.pone.0359352 (PMC13630237; doi:10.1371/journal.pone.0359352)
Supplement: Peer review history

# Peer review: Decision Letter 0

**DOI:** 10.1371/journal.pone.0359352.r001

**Authors:** Marco Innamorati

5 May 2026

Dear Dr. Ruiz,

Thank you for submitting your manuscript to PLOS ONE. After careful consideration, we feel that it has merit but does not fully meet PLOS ONE’s publication criteria as it currently stands. Therefore, we invite you to submit a revised version of the manuscript that addresses the points raised during the review process.

Please submit your revised manuscript by Jun 19 2026 11:59PM. If you will need more time than this to complete your revisions, please reply to this message or contact the journal office at plosone@plos.org. When you're ready to submit your revision, log on to https://www.editorialmanager.com/pone/ and select the 'Submissions Needing Revision' folder to locate your manuscript file.

- A letter that responds to each point raised by the academic editor and reviewer(s). You should upload this letter as a separate file labeled 'Response to Reviewers'.
- A marked-up copy of your manuscript that highlights changes made to the original version. You should upload this as a separate file labeled 'Revised Manuscript with Track Changes'.
- An unmarked version of your revised paper without tracked changes. You should upload this as a separate file labeled 'Manuscript'.

If you would like to make changes to your financial disclosure, please include your updated statement in your cover letter. Guidelines for resubmitting your figure files are available below the reviewer comments at the end of this letter.

If applicable, we recommend that you deposit your laboratory protocols in protocols.io to enhance the reproducibility of your results. Protocols.io assigns your protocol its own identifier (DOI) so that it can be cited independently in the future. For instructions see: https://journals.plos.org/plosone/s/submission-guidelines#loc-laboratory-protocols. Additionally, PLOS ONE offers an option for publishing peer-reviewed Lab Protocol articles, which describe protocols hosted on protocols.io. Read more information on sharing protocols at https://plos.org/protocols?utm_medium=editorial-email&utm_source=authorletters&utm_campaign=protocols.

As the corresponding author, your ORCID iD is verified in the submission system and will appear in the published article. PLOS supports the use of ORCID, and we encourage all coauthors to register for an ORCID iD and use it as well. Please encourage your coauthors to verify their ORCID iD within the submission system before final acceptance, as unverified ORCID iDs will not appear in the published article. *Only*  the individual author can complete the verification step; PLOS staff *cannot*  verify ORCID iDs on behalf of authors.

We look forward to receiving your revised manuscript.

Kind regards,

Marco Innamorati

Academic Editor

PLOS One

Journal requirements:

When submitting your revision, we need you to address these additional requirements.

1. Please ensure that your manuscript meets PLOS ONE's style requirements, including those for file naming. The PLOS ONE style templates can be found at

https://journals.plos.org/plosone/s/file?id=wjVg/PLOSOne_formatting_sample_main_body.pdf and

https://journals.plos.org/plosone/s/file?id=ba62/PLOSOne_formatting_sample_title_authors_affiliations.pdf

2. We noticed you have some minor occurrence of overlapping text with the following previous publication(s), which needs to be addressed:

- https://doi.org/10.1016/j.jcbs.2024.100854

- https://doi.org/10.1016/j.jcbs.2024.100854

In your revision ensure you cite all your sources (including your own works), and quote or rephrase any duplicated text outside the methods section. Further consideration is dependent on these concerns being addressed.

If the reviewer comments include a recommendation to cite specific previously published works, please review and evaluate these publications to determine whether they are relevant and should be cited. There is no requirement to cite these works unless the editor has indicated otherwise.

[Note: HTML markup is below. Please do not edit.]

Reviewers' comments:

Reviewer's Responses to Questions

**Comments to the Author**

1. Is the manuscript technically sound, and do the data support the conclusions?

Reviewer #1: Partly

Reviewer #2: Yes

**********

2. Has the statistical analysis been performed appropriately and rigorously?

Reviewer #1: Yes

Reviewer #2: Yes

**********

3. Have the authors made all data underlying the findings in their manuscript fully available?

The PLOS Data policy

Reviewer #1: Yes

Reviewer #2: Yes

**********

4. Is the manuscript presented in an intelligible fashion and written in standard English?

Reviewer #1: Yes

Reviewer #2: Yes

**********

Reviewer #1: **Recommendation: Major Revision**

This manuscript examines the psychometric properties of the AAQ-III and PsyFlex using network psychometric methods in a Colombian sample. The study is well executed and addresses an important issue in the ACT literature—namely, concerns about the discriminant validity of commonly used measures of psychological (in)flexibility. The use of EGA and bootEGA is technically sound, the sample size is adequate, and the overall pattern of results is coherent and consistent with theoretical expectations. The manuscript is clearly written and represents a potentially valuable contribution.

At the same time, several issues limit the strength of the conclusions and should be addressed before the manuscript can be considered for acceptance.

First, the claims regarding discriminant validity are overstated relative to the evidence provided. The separation of item communities in a network model is informative, but it is not equivalent to demonstrating discriminant validity in the conventional psychometric sense. This distinction is not sufficiently acknowledged, and the current framing risks implying stronger conclusions than the data support. The authors should temper these claims and clarify the specific inferences that can—and cannot—be drawn from the analytic approach used.

Second, the manuscript positions network psychometrics as a conceptually and methodologically preferable approach, but this claim is asserted more than demonstrated. Because no direct comparison is made with more conventional approaches (e.g., CFA or related latent variable models), it remains unclear whether the conclusions meaningfully differ from those obtained using standard methods. A more cautious and qualified presentation would strengthen the paper.

Third—and most importantly—the reporting of the measurement procedures, particularly regarding translation and adaptation, is insufficient and represents a substantive limitation. While the manuscript notes that translation followed general guidelines, it does not provide enough detail to evaluate the quality or rigor of the process. It remains unclear whether standard procedures such as forward–backward translation, expert panel review, or pilot testing were implemented, and how discrepancies were resolved. This is especially critical given that the central contribution of the manuscript is to provide validation evidence for Spanish versions of these instruments. Without a clear and transparent account of how the measures were translated and adapted, it is difficult to determine whether the observed psychometric properties reflect the intended constructs or artifacts of the translation process. In its current form, this lack of detail undermines confidence in the validity claims and should be addressed directly and thoroughly.

Finally, the broader claims about the AAQ-III resolving prior concerns with the AAQ-II extend beyond what can be supported by the current design. In the absence of direct comparative analyses, these conclusions should be framed more cautiously.

In sum, this is a solid and promising study, but the manuscript would benefit from a tighter alignment between the analytic approach, the methodological transparency, and the scope of the claims. In particular, clarifying and strengthening the description of the translation and adaptation procedures is essential. Addressing these points should substantially improve the manuscript and clarify its contribution.

Reviewer #2: I would like to thank the editors of PLOS ONE for the opportunity to review this manuscript. I found it very interesting and enjoyed reading it. The manuscript is clear, concise, and well-written, and the analyses are accurate. I believe it will be of great interest to the journal readership and recommend its publication. However, I would like to comment on a few points that caught my attention and suggest some minor changes to the authors that, in my opinion, will help the reader better understand the novelty and implications of this study’s results within the context of the literature on psychological flexibility and inflexibility.

From the very beginning of the manuscript, the authors clearly position themselves by assuming that psychological flexibility and inflexibility are related but independent constructs and not part of a continuum. Thus, as early as the second paragraph, they state, “This evidence indicates that psychological flexibility and inflexibility are related but distinct functional patterns rather than exact opposites.” In my view, this categorical statement should be qualified, since, as the authors later acknowledge, the most widely used instrument in research on psychological flexibility and inflexibility has been the AAQ-II, a unifactorial measure that has been used interchangeably as an instrument of flexibility, inflexibility, or experiential avoidance, simply by changing the sign of the scores (an example can be seen in the work by Benoy et al. cited on page 6 of the introduction). To conclude that these are two distinct processes based on the limited research conducted with the Multidimensional Psychological Flexibility Inventory (MPFI)—which does indeed include independent scales and allows for differentiated analyses—seems somewhat risky to me. Furthermore, assuming from the outset that the evidence in this regard is solid somehow detracts from the significance of the authors’ own findings.

Aside from this, I find the introduction lacking a slightly more detailed description of the previous validation studies of the AAQ-III and Psyflex instruments.

In the Methods section, I would appreciate that the researchers could describe the process of translating the instruments in somewhat greater detail, given its obvious relevance.

In the results section, regarding the correlation matrix presented in Figure 5, I would ask the authors to include the significance of the correlations. On the other hand, although the color coding in the table distinguishing between positive and negative correlations allows for quick reference, the darker cells make it difficult to read, so I leave it to the editor’s discretion whether or not to retain the color coding.

Finally, I found the discussion lacking in an analysis of certain results that I consider relevant. The results of the EGA conducted using the AAQ-III, Psyflex, DASS-21, and BFI-2-Neuroticism clearly show that we are dealing with different dimensions, which would support the idea that the AAQ-III is not a measure of negative emotion or neuroticism. However, it is striking to observe in the correlation matrix that the AAQ-III shows a stronger correlation with the DASS-21 than with the inflexibility scale of the MPFI-SF and its various factors (which were not included in the EGA analyses). There is no mention of this in the discussion, and it might warrant some comment.

On the other hand, the positive correlation found between the AAQ-III and the DASS-21 is stronger than the negative correlation found between the Psyflex and the DASS-21, which would support the authors’ view that both instruments measure related, but not opposite, processes. However, the magnitude of the correlations between both instruments (AAQ-III and Psyflex) and the Satisfaction with Life Scale (SWLS) is similar, which, in a way, contradicts the proposal, and this finding is not discussed.

I would encourage the authors to address these highly interesting results in the discussion, with a view to a conceptual analysis of psychological flexibility and inflexibility as measured by the AAQ-III and the Psyflex.

Finally, I would ask the authors to correct the volume number in the references for the article by Benoy et al. (2019), which was published in volume 13, not 12.

I would like to reiterate that this is a relevant and rigorous article and point out that my comments are in no way intended to correct the authors’ work, but rather to offer some ideas that might improve the manuscript.

**********

what does this mean?). If published, this will include your full peer review and any attached files.

If you choose “no”, your identity will remain anonymous but your review may still be made public.

**Do you want your identity to be public for this peer review?**  For information about this choice, including consent withdrawal, please see our Privacy Policy

Reviewer #1: **Yes:**  Antonio Cepeda-Benito

Reviewer #2: No

**********

[NOTE: If reviewer comments were submitted as an attachment file, they will be attached to this email and accessible via the submission site. Please log into your account, locate the manuscript record, and check for the action link "View Attachments". If this link does not appear, there are no attachment files.]

To ensure your figures meet our technical requirements, please review our figure guidelines: https://journals.plos.org/plosone/s/figures

You may also use PLOS’s free figure tool, NAAS, to help you prepare publication quality figures: https://journals.plos.org/plosone/s/figures#loc-tools-for-figure-preparation.

NAAS will assess whether your figures meet our technical requirements by comparing each figure against our figure specifications.

# Author comment: Author response to Decision Letter 1

**DOI:** 10.1371/journal.pone.0359352.r002

21 Jul 2026

Response to Reviewers

Manuscript: PONE-D-26-09970

Title: A network psychometric evaluation of the Acceptance and Action Questionnaire – III and PsyFlex

Dear Dr. Innamorati,

We thank you and the two reviewers for the careful and constructive evaluation of our manuscript. The comments have helped us substantially improve the clarity of our framing, the transparency of our methodological reporting, and the calibration of our inferential claims. We have prepared a revised version that addresses each point raised and includes detailed responses to all reviewers' comments.

Regarding the additional journal requirements, we have reformatted the manuscript in accordance with the PLOS ONE style templates. With respect to the textual overlap with one of our previous works, we identified the affected passages and have rephrased them, incorporating the appropriate citation to that work.

A point-by-point response to the editorial requirements and to the reviewers' comments follows below. Changes in the manuscript are described in the corresponding responses, and a marked-up version is provided as a separate file.

Additional changes: Recently published evidence on the Spanish PsyFlex. We have found two recently published studies examining Spanish versions of the PsyFlex: Navarrete et al. (2025), who analyzed a different Spanish translation in individuals with chronic pain, and Crego et al. (2026), who analyzed the same version used in the current study in a sample of 170 healthy individuals. The Introduction, Procedure, and Discussion have been updated to acknowledge this emerging evidence and to position the current study accordingly (as the first examination of the PsyFlex in a large general-population sample from Latin America and the first psychometric analysis of the Spanish AAQ-III).

Reviewer 1

Reviewer comment 1. The claims regarding discriminant validity are overstated relative to the evidence provided. The separation of item communities in a network model is informative, but it is not equivalent to demonstrating discriminant validity in the conventional psychometric sense. This distinction is not sufficiently acknowledged, and the current framing risks implying stronger conclusions than the data support. The authors should temper these claims and clarify the specific inferences that can—and cannot—be drawn from the analytic approach used.

Author response. We thank the reviewer for this observation, with which we essentially agree. The separation of item communities in a joint EGA indicates that the items of the AAQ-III and the PsyFlex share more unique variance among themselves than with indicators of emotional distress and neuroticism. This is structural, item-level evidence, and it is not equivalent to demonstrating discriminant validity in the conventional latent-variable sense. We have revised the manuscript to make this distinction explicit and to temper the corresponding claims: (a) the Abstract now states that the joint EGA provided "structural evidence consistent with the discriminant validity of both measures"; (b) the Data analysis section now clarifies that the separation of communities "provides item-level evidence consistent with discriminant validity, although this criterion is not equivalent to conventional tests based on latent variable models"; (c) the Discussion sentence stating that the findings "confirm that the AAQ-III provides superior discriminant validity compared to the AAQ-II" now indicates that the findings are consistent with previous evidence, and we explicitly acknowledge that this comparison is indirect given that the AAQ-II was not administered in the current study; and (d) a new limitation specifies the inferences that can and cannot be drawn from the joint EGA.

Additionally, we computed heterotrait-monotrait ratios (HTMT; Henseler et al., 2015) as a conventional check of discriminant validity. All ratios were below the conservative .85 threshold: .54 for the AAQ-III and the DASS-21, .38 for the PsyFlex and the DASS-21, and .56 for the PsyFlex and neuroticism. The HTMT between the AAQ-III and neuroticism was .83, below the threshold but close to it, which indicates that the differentiation between psychological inflexibility and neuroticism, although present, is not large. This result is consistent with the more cautious framing now adopted throughout the manuscript. We did not include these analyses in the manuscript to keep its focus on the network approach, but we would be glad to add them as supporting information if the reviewer or the editor considers it appropriate.

Reviewer comment 2. The manuscript positions network psychometrics as a conceptually and methodologically preferable approach, but this claim is asserted more than demonstrated. Because no direct comparison is made with more conventional approaches (e.g., CFA or related latent variable models), it remains unclear whether the conclusions meaningfully differ from those obtained using standard methods. A more cautious and qualified presentation would strengthen the paper.

Author response. We agree that the original wording positioned network psychometrics as preferable in a way that our design does not directly test, and we have revised the framing accordingly: (a) the Introduction no longer claims that EGA provides researchers with fewer degrees of freedom in absolute terms; it now notes that the algorithm is deterministic once the estimation method and the community detection algorithm have been specified, and it states explicitly that we regard network psychometrics as a complement to factor-analytic approaches rather than as a substitute for them; (b) in the Data analysis section, "EGA has some notable advantages over traditional factor analytic methods" now reads "EGA differs from traditional factor analytic methods in several respects," and the only comparative claim retained (accuracy in dimensionality recovery) is the one supported by the simulation studies cited; and (c) a new limitation acknowledges that this study did not directly compare EGA with latent variable models and that our conclusions should be interpreted as complementary to evidence derived from such approaches.

Regarding whether the conclusions would meaningfully differ from those obtained with standard methods, we conducted confirmatory factor analyses as a check (robust DWLS estimation; both models included correlated residuals between semantically related item pairs), although we did not include them in the manuscript. The unidimensional models converged with the EGA solutions. The AAQ-III showed excellent fit, CFI = .999, TLI = .999, RMSEA = .021 (90% CI [.000, .043]), SRMR = .029, and the PsyFlex showed adequate fit, CFI = .991, TLI = .982, SRMR = .047, with a somewhat elevated RMSEA of .074 (90% CI [.051, .098]), which is common in models with few degrees of freedom. Reliability was adequate for both measures (omega = .93 and .81, respectively). These results suggest that the substantive conclusions regarding dimensionality do not depend on the analytic approach. We would also be glad to report these analyses as supporting information if deemed useful.

Reviewer comment 3. Most importantly—the reporting of the measurement procedures, particularly regarding translation and adaptation, is insufficient and represents a substantive limitation. While the manuscript notes that translation followed general guidelines, it does not provide enough detail to evaluate the quality or rigor of the process. It remains unclear whether standard procedures such as forward–backward translation, expert panel review, or pilot testing were implemented, and how discrepancies were resolved. This is especially critical given that the central contribution of the manuscript is to provide validation evidence for Spanish versions of these instruments. Without a clear and transparent account of how the measures were translated and adapted, it is difficult to determine whether the observed psychometric properties reflect the intended constructs or artifacts of the translation process. In its current form, this lack of detail undermines confidence in the validity claims and should be addressed directly and thoroughly.

Author response. We agree that the reporting of the translation and adaptation procedures was insufficient, and we have expanded the Procedure section to describe them in detail. The revised text specifies the following. The translation of the AAQ-III followed a forward translation procedure with expert panel reconciliation: two ACT researchers with bilingual proficiency in Spanish and English independently translated the seven items, using as the starting point the Spanish version of the AAQ-II developed by Ruiz et al. [35], which has accumulated evidence of construct validity in Colombia and other Spanish-speaking countries [17,34]. This decision was made given that the AAQ-III revised the wording of the AAQ-II items rather than introducing entirely new content, which allowed translators to preserve semantic continuity for the items whose meaning had not changed and to concentrate on the functional specifications introduced in the AAQ-III. The two independent translations were subsequently reviewed by a panel of four researchers with expertise in ACT and CBS, who discussed each item until consensus was reached on the version that best preserved the semantic content, the functional specification, and the cultural appropriateness of each item.

Regarding the PsyFlex, this study did not generate a new translation. The starting point was the Spanish version previously developed by members of our research team for the international study on the mental health impact of the COVID-19 pandemic conducted by Gloster et al. (2020), now cited in the manuscript [49]. Although this version had been used in previous research, its psychometric properties had not been formally examined in Spanish-speaking samples. For this study, the same expert panel reviewed each item against the original English wording and introduced modifications in those items whose previous wording could be improved in terms of fluency, semantic precision, or alignment with the hexaflex process represented by the item. The final Spanish wording of all items, alongside the original English versions, is shown in Tables 1 and 2.

The Discussion acknowledges that (a) formal back-translation by an independent bilingual translator was not implemented, given that the two independent forward translations for the AAQ-III and the expert panel review for both instruments were intended to accomplish complementary functions, although they do not fully substitute for back-translation; and (b) the items were not subjected to cognitive interviewing with members of the target population before administration. Both procedures are recommended in the guidelines for test translation and adaptation and should be incorporated in future cross-cultural validation work. However, we think the observed psychometric properties are unlikely to reflect mere translation artifacts: the network loadings, the stability results, and the pattern of correlations with the remaining instruments were highly consistent with those reported for the original English versions, which would not be expected if the item content had been substantially distorted in translation.

Reviewer comment 4. The broader claims about the AAQ-III resolving prior concerns with the AAQ-II extend beyond what can be supported by the current design. In the absence of direct comparative analyses, these conclusions should be framed more cautiously.

Author response. We agree with the reviewer. As noted in our response to Comment 1, the sentence claiming that the findings confirmed the AAQ-III's superior discriminant validity has been revised, and the Discussion now explicitly acknowledges that the AAQ-II was not administered in the current study and that comparisons with it are indirect. In line with the present comment, we have made three additional changes: (a) the Discussion now describes the results as providing initial (rather than strong) evidence supporting the internal structure, stability, and validity of both measures; (b) the contrast with the AAQ-II is now explicitly framed as a comparison with patterns reported in previous studies; and (c) the limitations section now states that this study did not include the AAQ-II, which precludes direct comparative analyses between the AAQ-III and its predecessor. Claims about the AAQ-III resolving the concerns raised about the AAQ-II would require head-to-head comparisons in the same sample, and we have been careful to avoid implying that the current design provides them.

Response to Reviewer 2

Reviewer comment 1. From the very beginning of the manuscript, the authors clearly position themselves by assuming that psychological flexibility and inflexibility are related but independent constructs and not part of a continuum. Thus, as early as the second paragraph, they state, "This evidence indicates that psychological flexibility and inflexibility are related but distinct functional patterns rather than exact opposites." In my view, this categorical statement should be qualified, since, as the authors later acknowledge, the most widely used instrument in research on psychological flexibility and inflexibility has been the AAQ-II, a unifactorial measure that has been used interchangeably as an instrument of flexibility, inflexibility, or experiential avoidance, simply by changing the sign of the scores (an example can be seen in the work by Benoy et al. cited on page 6 of the introduction). To conclude that these are two distinct processes based on the limited research conducted with the Multidimensional Psychological Flexibility Inventory (MPFI)—which does indeed include independent scales and allows for differentiated analyses—seems somewhat risky to me. Furthermore, assuming from the outset that the evidence in this regard is solid somehow detracts from the significance of the authors' own findings.

Author response. We agree that the sentence was too categorical, and we appreciate the reviewer's observation that presenting this evidence as settled detracts from the current study's contribution. We have qualified the statement in the second paragraph of the Introduction, which now indicates that the available evidence suggests that both constructs might be related but distinct functional patterns. We have also added an explicit acknowledgment that most of this evidence comes from studies using multidimensional instruments that assess flexibility and inflexibility processes via separate scales (such as the MPFI), and that the degree of overlap between the two constructs when measured with brief, unidimensional instruments remains an open question. Accordingly, the Discussion now frames our findings (the moderate-to-high correlation between the AAQ-III and the PsyFlex, their differential patterns of association with related constructs, and their separation into distinct communities) as evidence that informs this question rather than as confirmation of a settled matter.

Reviewer comment 2. Aside from this, I find the introduction lacking a slightly more detailed description of the previous validation studies of the AAQ-III and Psyflex instruments.

Author response. We have expanded the second and third paragraphs of the Introduction addressing the AAQ-III and the PsyFlex. Regarding the AAQ-III, the revised text now describes the design of the original psychometric comparison by Ong et al. [24] (measures compared, samples, and analytic approach) and specifies its main findings concerning discriminant validity. We have also expanded the description of the Chinese validation of the AAQ-3 [26], which now details the sample, the replication of the one-factor structure, its internal consistency, and its measurement invariance across gender. Regarding the PsyFlex, the sentence that merely listed the available cross-cultural validations has been expanded to describe the specific contribution of each study (Portuguese, Korean, Turkish, Hebrew, and Chinese versions). We opted for a concise expansion to keep the Introduction focused, but we would be glad to provide additional detail if the reviewer considers it necessary.

Reviewer comment 3. In the Methods section, I would appreciate that the researchers could describe the process of translating the instruments in somewhat greater detail, given its obvious relevance.

Author response. Please, see our response to Comment 3 from Reviewer #1.

Reviewer comment 4. In the results section, regarding the correlation matrix presented in Figure 5, I would ask the authors to include the significance of the correlations. On the other hand, although the color coding in the table distinguishing between positive and negative correlations allows for quick reference, the darker cells make it difficult to read, so I leave it to the editor's discretion whether or not to retain the color coding.

Author response. While revising Fig 5 to add the significance of the correlations, we detected and corrected a computation error in the committed action subscale of the MPFI-SF. The corrected correlation between the AAQ-III and the PsyFlex is r = -.38 (previously reported as -.56), and the corresponding sentences in the Results and Discussion have been updated. This correction does not affect any other result: the EGA, bootEGA, and joint EGA analyses did not include the MPFI-SF, and the remaining correlations in Fig 5 were unaffected. If anything, the corrected value strengthens the conclusion that the AAQ-III and the PsyFlex are not interchangeable.

Reviewer comment 5. Finally, I found the discussion lacking in an analysis of certain results that I consider relevant. The results of the EGA conducted using the AAQ-III, Psyflex, DASS-21, and BFI-2-Neuroticism clearly show that we are dealing with different dimensions, which would support the idea that the AAQ-III is not a measure of negative emotion or neuroticism. However, it is striking to observe in the correlation matrix that the AAQ-III shows a stronger correlation with the DASS-21 than with the inflexibility scale of the MPFI-SF and its various factors (which were not included in the EGA analyses). There is no mention of this in the discussion, and it might warrant some comment. On the other hand, the positive correlation found between the AAQ-III and the DASS-21 is stronger than the negative correlation found between the Psyflex and the DASS-21, which would support the authors' view that both instruments measure related, but not opposite, processes. However, the magnitude of the correlations between both instruments (AAQ-III and Psyflex) and the Satisfaction with Life Scale (SWLS) is similar, which, in a way, contradicts the proposal, and this finding is not discussed. I would encourage the authors to address these highly interesting results in the discussion, with a view to a conceptual analysis of psychological flexibility and inflexibility as measured by the AAQ-III and the Psyflex.

Author response. We thank the reviewer for this thoughtful analysis, which we have incorporated into the Discussion. Two new paragraphs now address the patterns highlighted by the reviewer.

First, regarding the stronger correlation of the AAQ-III with the DASS-21 (r = .72) than with the inflexibility composite of the MPFI-SF (r = .61) and its factors, we suggest that this pattern reflects (a) the item content of the AAQ-III, which emphasizes the interference of distressing private experiences with valued living and thus shares affective content with symptom measures that is less present in the process-specific wording of the MPFI-SF; and (b) the conceptualization of psychological inflexibility as a process directly involved in generating and maintaining emotional distress, which makes strong correlations with symptom measures theoretically expected. We also note that the joint EGA indicates that this overlap does not entail item-level redundancy, given that the AAQ-III items formed a community clearly separated from emotional symptoms and neuroticism. In addition, the Discussion now comments on the near-zero correlation of the AAQ-III with the experiential avoidance factor of the MPFI-SF, which also showed positive correlations with flexibility processes. This divergent behavior is consistent with the theoretically inconsistent correlations that this factor has shown in previous validations of the MPFI, which have been identified as one of the clear weaknesses of the instrument (Hekmati et al., 2024; Navarrete et al., 2025; Tabrizi et al., 2022; now cited as references 59–61).

Second, we agree that the asymmetry between the correlations of the AAQ-III (r = .72) and the PsyFlex (r = -.34) with the DASS-21 supports the view that both instruments measure related but not opposite processes, and this is now stated explicitly. Third, we also agree that the similar magnitudes of the correlations between both instruments and the SWLS (rs = -.46 and .46) support this differential pattern, and this finding is now discussed rather than omitted. The Discussion acknowledges that the characterization of the PsyFlex as a marker of protective resources and the AAQ-III as an indicator of transdiagnostic vulnerability applies more clearly to symptom measures than to global well-being indicators, offers a plausible interpretation (life satisfaction judgments are sensitive both to the presence of emotional distress and to engagement in valued living, so that both poles contribute to them through different paths), and identifies these differential patterns as a target for future studies including measures of valued living and positive functioning beyond global life satisfaction.

Reviewer comment 6. Finally, I would ask the authors to correct the volume number in the references for the article by Benoy et al. (2019), which was published in volume 13, not 12.

Author response. Thanks for noting. We have corrected this.

Attachment Submitted filename: Response Letter 14-07.docx

# Peer review: Decision Letter 1

**DOI:** 10.1371/journal.pone.0359352.r003

**Authors:** Marco Innamorati

13 Sep 2026

A network psychometric evaluation of the Acceptance and Action Questionnaire – III and PsyFlex

PONE-D-26-09970R1

Dear Dr. Ruiz,

We’re pleased to inform you that your manuscript has been judged scientifically suitable for publication and will be formally accepted for publication once it meets all outstanding technical requirements.

Within one week, you’ll receive an e-mail detailing the required amendments. When these have been addressed, you’ll receive a formal acceptance letter and your manuscript will be scheduled for publication.

An invoice will be generated when your article is formally accepted. Please note, if your institution has a publishing partnership with PLOS and your article meets the relevant criteria, all or part of your publication costs will be covered. Please make sure your user information is up-to-date by logging into Editorial Manager at Editorial Manager® and clicking the ‘Update My Information' link at the top of the page. For questions related to billing, please contact billing support.

If your institution or institutions have a press office, please notify them about your upcoming paper to help maximize its impact. If they’ll be preparing press materials, please inform our press team as soon as possible -- no later than 48 hours after receiving the formal acceptance. Your manuscript will remain under strict press embargo until 2 pm Eastern Time on the date of publication. For more information, please contact onepress@plos.org.

Kind regards,

Marco Innamorati

Academic Editor

PLOS One

Additional Editor Comments (optional):

Dear Authors,

when correcting the proofs, please include corrections suggested by one of the reviewer: "Two minor wording inconsistencies remain. In the Results, the statement that the findings “strongly support the discriminant validity” of the AAQ-III and PsyFlex should be revised to indicate that they provide “structural evidence consistent with discriminant validity,” in keeping with the more appropriately qualified language used elsewhere in the manuscript. Similarly, the Conclusion’s reference to “these validated measures” should be changed to “these measures, for which the present study provides initial validation evidence,” or comparable wording."

Reviewers' comments:

Reviewer's Responses to Questions

**Comments to the Author**

Reviewer #1: All comments have been addressed

Reviewer #2: All comments have been addressed

**********

2. Is the manuscript technically sound, and do the data support the conclusions?

Reviewer #1: Yes

Reviewer #2: Yes

**********

3. Has the statistical analysis been performed appropriately and rigorously?

Reviewer #1: Yes

Reviewer #2: Yes

**********

4. Have the authors made all data underlying the findings in their manuscript fully available?

The PLOS Data policy

Reviewer #1: Yes

Reviewer #2: Yes

**********

5. Is the manuscript presented in an intelligible fashion and written in standard English?

Reviewer #1: Yes

Reviewer #2: Yes

**********

Reviewer #1: Thank you for the careful and comprehensive revisions. The authors have addressed my principal concerns.

Two minor wording inconsistencies remain. In the Results, the statement that the findings “strongly support the discriminant validity” of the AAQ-III and PsyFlex should be revised to indicate that they provide “structural evidence consistent with discriminant validity,” in keeping with the more appropriately qualified language used elsewhere in the manuscript. Similarly, the Conclusion’s reference to “these validated measures” should be changed to “these measures, for which the present study provides initial validation evidence,” or comparable wording.

Subject to these minor corrections, I believe the manuscript is suitable for publication.

Reviewer #2: The authors have addressed all the points that were brought to their attention, and in my opinion, the paper, in its current form, is ready for publication.

**********

what does this mean?). If published, this will include your full peer review and any attached files.

If you choose “no”, your identity will remain anonymous but your review may still be made public.

**Do you want your identity to be public for this peer review?**  For information about this choice, including consent withdrawal, please see our Privacy Policy

Reviewer #1: **Yes:**  Antonio Cepeda-Benito

Reviewer #2: No

**********

# Editor report: Acceptance letter

**DOI:** 10.1371/journal.pone.0359352.r004

**Authors:** Marco Innamorati

PONE-D-26-09970R1

PLOS One

Dear Dr. Ruiz,

I'm pleased to inform you that your manuscript has been deemed suitable for publication in PLOS One. Congratulations! Your manuscript is now being handed over to our production team.

At this stage, our production department will prepare your paper for publication. This includes ensuring the following:

* All references, tables, and figures are properly cited

* All relevant supporting information is included in the manuscript submission,

* There are no issues that prevent the paper from being properly typeset

You will receive further instructions from the production team, including instructions on how to review your proof when it is ready. Please keep in mind that we are working through a large volume of accepted articles, so please give us a few days to review your paper and let you know the next and final steps.

Lastly, if your institution or institutions have a press office, please let them know about your upcoming paper now to help maximize its impact. If they'll be preparing press materials, please inform our press team within the next 48 hours. Your manuscript will remain under strict press embargo until 2 pm Eastern Time on the date of publication. For more information, please contact onepress@plos.org.

You will receive an invoice from PLOS for your publication fee after your manuscript has reached the completed accept phase. If you receive an email requesting payment before acceptance or for any other service, this may be a phishing scheme. Learn how to identify phishing emails and protect your accounts at https://explore.plos.org/phishing.

If we can help with anything else, please email us at customercare@plos.org.

Thank you for submitting your work to PLOS One and supporting open access.

Kind regards,

PLOS One Editorial Office Staff

on behalf of

Dr. Marco Innamorati

Academic Editor

PLOS One
